# Enhancing Emotional Intelligence in Autism Spectrum Disorder Through Intervention: A Systematic Review

**DOI:** 10.3390/ejihpe15030033

**Published:** 2025-03-10

**Authors:** Laura García-García, Manuel Martí-Vilar, Sergio Hidalgo-Fuentes, Javier Cabedo-Peris

**Affiliations:** 1 Basic Psychology Department, Faculty of Psychology and Speech Therapy, Universitat de València, 46010 Valencia, Spain; laugar33@alumni.uv.es (L.G.-G.); sergio.hidalgo@uv.es (S.H.-F.); 2 Faculty of Health Sciences, Universidad Internacional de Valencia (VIU), 46002 Valencia, Spain; javier.cabedo@professor.universidadviu.com

**Keywords:** autism spectrum disorder, emotional intelligence, emotional regulation, emotional recognition ability

## Abstract

Limitations in some emotional characteristics that are conceptualized in the definition of emotional intelligence can be seen among people with autism spectrum disorder. The main objective of this study is the analysis of the effectiveness of interventions directed to enhance emotional recognition and emotional regulation among this specific population. A systematic review was carried out in databases such as Psycinfo, WoS, SCOPUS, and PubMed, identifying a total of 572 articles, of which 29 met the inclusion criteria. The total sample included 1061 participants, mainly children aged between 4 and 13 years. The analyzed interventions focused on improving emotional recognition, with significant results in the identification of emotions such as happiness, sadness, and anger, although some showed limitations in the duration of these effects. The most used programs included training in facial recognition, virtual reality, and the use of new technologies such as robots. These showed improvements in both emotional recognition and social skills. Other types of interventions such as music therapy or the use of drama techniques were also implemented. However, a gender bias and lack of consistency between results from different cultures were observed. The conclusions indicate that, although the interventions reviewed seem effective, more research is needed to maximize their impact on the ASD population.

## 1. Introduction

According to the World Health Organization (WHO, 2023), it is estimated that approximately one in one-hundred children worldwide has autism spectrum disorder (ASD). Over the past decades, an increase in the prevalence of ASD has been observed (Sevilla et al., 2013) and occurs in 1% of the global population (Zeidan et al., 2022). Therefore, studying ASD is crucial to ensuring an optimal quality of life for individuals and improving their personal development.

### 1.1. Autism Spectrum Disorder (ASD)

ASD is defined as a group of conditions characterized by difficulties in social interaction and communication (WHO, 2023). The Diagnostic and Statistical Manual of Mental Disorders (DSM-5-TR) describes autism as a neurodevelopmental disorder manifested by deficits in communication and social interaction, as well as restrictive and repetitive behavioral patterns (American Psychological Association (APA), 2022). The term autism was first coined by Kanner in 1943 and was applied to children with atypical socio-emotional development and significant socialization problems (Kanner, 1946). However, autism was not yet considered a specific diagnosis but rather a characteristic of childhood schizophrenia (Alcalá & Ochoa-Madrigal, 2022). It was not until the publication of the DSM-III-R (American Psychological Association, 1987) that it was named “Autistic Disorder.” Subsequent editions and modifications of the manual led to interpreting autism as a spectrum due to its etiology. To this day, the etiology remains unknown, and it is attributed to multifactorial reasons.

ASD symptoms are varied and usually manifest around 18 months, becoming more consolidated by 36 months of age (Reynoso et al., 2017). ASD is considered a spectrum, although there are common characteristics that form the basis for diagnosis. These characteristics are, for example, complex cognitive abilities developed from the first year of life, which are part of the theory of mind (ToM). People with ASD demonstrate a deficit in this capacity, which prevents them from attributing mental states to themselves and others (Hernández-Núñez, 2018). The ToM refers to the system humans use to infer mental states through behavior (Baron-Cohen et al., 1995). For instance, this includes the ability to predict others’ behavior, feelings, and intentions (Tirapu-Ustárroz et al., 2007). Considering that, their social relationships and development are significantly limited, hindering successful personal development.

ASD is often associated with other neurodevelopmental disorders (Lai et al., 2014). Historically, there has been an interest in studying the relationship between ASD and intelligence, often associating ASD with the intelligence quotient (IQ), as Binet and Simon did (Mora & Martín, 2007), or with a direct relationship with intellectual disability ([ID] López et al., 2009). As previously mentioned, ID is not a diagnostic determinant, although it may be comorbid. In fact, ID can occur in all variations of the spectrum except for Asperger’s syndrome or high-functioning ASD. Thus, considering the entire spectrum, two types of disorders can be identified: low-functioning ASD or ASD with ID and high-functioning ASD or Asperger’s syndrome (Vargas et al., 2019).

### 1.2. ASD and Emotional Intelligence (EI)

Emotional intelligence (EI) can be defined as encompassing all processes involved in recognizing, using, understanding, and managing emotional states, both one’s own and those of others (J. D. Mayer et al., 2008; Salovey & Mayer, 1990). Consequently, EI is understood as a set of multiple abilities, including the following: (a) emotional perception and expression, (b) emotional understanding, (c) emotional regulation (ER), and (d) the appropriate management and integration of emotions into reasoning. In fact, J. D. Mayer et al. (2000) developed a model of EI, stating that this construct comprises four components corresponding to distinct skills that influence our environment and social relationships. These skills include the following: (a) the ability to accurately perceive emotions, (b) the capacity to leverage emotional information to facilitate other cognitive processes, (c) the ability to understand emotions, and (d) the ability to regulate one’s own emotions as well as those of others. This model is known as the Ability Model.

There are other authors that defined EI. For example, Goleman’s (1995) mixed model is focused on interpersonal and intrapersonal intelligence. It emphasizes its link to the human capacity to recognize, control, and understand emotions, and self-motivate and manage social relationships (Hernández-Núñez, 2018). This framework aligns with the construct of EI defined by Salovey and Mayer in the 1990s (Kanesan & Fauzan, 2019). Another example is Gardner’s (1983) multiple intelligences theory, which described several modular intelligences that interact while also operating independently (Fernández, 2019; Sharma & Pandey, 2024). Goleman agreed with Gardner in rejecting the notion of rational intelligence as the sole predictor of success (Barrera-Gálvez et al., 2019). Finally, Bar-On’s (2006) Traits Model focuses on self-perception and emotional skills. However, the Ability Model can be considered highly suitable for the study of ASD due to its features (Kanesan & Fauzan, 2019). On the one hand, it is considered the most scientifically rigorous. On the other, its very definition emphasizes the cognitive processing of emotional information, which is highly related to ASD (Aguaded Gómez & Valencia, 2017; Anderson, 2015; S. S. Kuo et al., 2019).

### 1.3. The Current Study

As previously noted, individuals with ASD exhibit deficits in fundamental cognitive functions that are part of the ToM. They demonstrate difficulties in competencies such as emotional recognition ability (ERA) on the self or others, ER, and socio-communicative interaction in general (Hernández-Núñez, 2018). These competencies are directly linked to the construct of EI defined by Salovey and Mayer (1990). The emotional needs of these individuals are constrained due to deficits in these executive functions, which significantly affect their developmental trajectory. ERA, among other skills, is essential for understanding the mood of others (Beall et al., 2008). Therefore, it is imperative to intervene with ASD individuals to help them acquire these skills.

Multiple and different types of therapies have been implemented among people with ASD (Antshel & Russo, 2019; Genovese & Butler, 2020; Ona et al., 2020; Urinovsky & Cafiero, 2022). Indeed, current research indicates that certain interventions for ASD show promising results, particularly in improving ER, which can be valuable for enhancing EI (Guz et al., 2024; Omelchenko et al., 2024; Salimi et al., 2019; White et al., 2009).

This observation gives rise to the Patients/Problem, Intervention, Comparison, and Outcomes (PICO) question. P stands for people with ASD, I stands for interventions designed to enhance EI, C stands for comparing the intervention with a control group, and O stands for analyzing the effectiveness of their results. This leads to the formulation of the following Research Question (RQ): How effective are the interventions designed to enhance EI in people with ASD? From this inquiry emerges the primary aim of this study: to gather information on the most suitable interventions for developing EI in individuals with ASD. Also, the second objective is to explore the characteristics of these interventions, to see which are the most effective.

The interventions are analyzed based on the facets of EI described by J. D. Mayer et al. (2008): (1) emotion perception, (2) emotional understanding, (3) emotional management, and (4) the use of emotions to facilitate thought. By examining EI through its individual components, this review aims to achieve a deeper understanding of the specific needs of these individuals.

## 2. Materials and Methods

The PICO question components were taken into account when conducting the systematic review. Additionally, the preferred reported items for systematic reviews and meta-analyses (PRISMA) guidelines (Page et al., 2021) were applied to ensure the proper execution of the systematic review. The different phases of the procedure are detailed below (Appendix A). The international prospective register of systematic reviews (PROSPERO) was used to preregister this process, with the identification number CRD42024587433.

### 2.1. Search Strategy and Sources of Information

A comprehensive analysis of available studies across various information sources was conducted to gather the most relevant evidence on the topic and adequately evaluate the established objectives. The systematic search was carried out between 10 April and 12 June 2024. The databases used included Psycinfo, Web of Science (WoS), SCOPUS, and PubMed, focusing on peer-reviewed scientific articles and excluding book chapters, dissertations, or editorials. The search terms used across all databases were as follows:

(Autis*) AND (“Emotional intelligence”) AND (Intervention).

The search was conducted on titles, abstracts, and keywords, without any time restrictions. It covered the period from 1980 to 2023, although no relevant records were found prior to 1984. It was considered not appropriate to include registers that were published after 2023, as the year 2024 was not finished when the review was completed. The term EI was used as a generic term to extend the results to the largest possible number of studies, leaving aside the components of its definition. However, the presence of these components was an essential criterium when the records identified from the databases were screened.

### 2.2. Inclusion and Exclusion Criteria

The selection process was based on the following criteria. Documents were included when they (a) were written in English or Spanish; (b) were empirical research articles, including both qualitative and quantitative scientific studies; (c) involved interventions related to the construct of EI, regarding the definition of J. D. Mayer et al. (2008), which covered all processes involved in recognizing, using, understanding, and managing emotional states, both one’s own and those of others; and (d) focused on individuals with ASD.

As exclusion criteria, the following were considered: (a) studies published after 31 December 2023; (b) book chapters, manuals, systematic reviews, meta-analyses, dissertations, editorials, or observational studies; (c) interventions not focused on ERA, regulation, or understanding; (d) interventions applied to parents or caregivers of individuals with ASD; (e) reports that focused mainly on the development of ER, rather than on their effectiveness; and (f) research which was not published in an open-access format and which could not be found by other means.

### 2.3. Codification and Critical Appraisal

These articles were analyzed in two stages: first, by reviewing only the title and abstract, and second, by reading the full text. During the review process, the Covidence platform was used to help with the management and organization of the literature review process by warranting the suitability of the studies. This tool ensured greater rigor and objectivity in the selection of studies, as each article was independently evaluated through an inter-rater examination. The following sections were extracted from each of the articles that were included in the systematic review. First, the authorship, year, and country of publication were extracted. Secondly, the main objective of studies with aspects related to EI was extracted. Third, regarding methodology aspects, the sample size, its division by sex, the number of participants with ASD, the presence of a control group, the type of intervention, its duration, and the evaluation instruments used were extracted. The reliability of the assessment tools was also reported when this was included in the original studies. Also, possible comorbidities between ASD and other disorders presented in the studies’ samples were reported, but only when the researchers explicitly indicated this in the body of the text. Finally, the results related to the objective of the present study were extracted.

To assess the quality of the selected studies, the Grading of Recommendations Assessment, Development, and Evaluation (GRADE) system was used. This framework determines the level of scientific evidence and the strength of recommendations for each study.

## 3. Results

### 3.1. Selection of Studies

**Figure 1:**
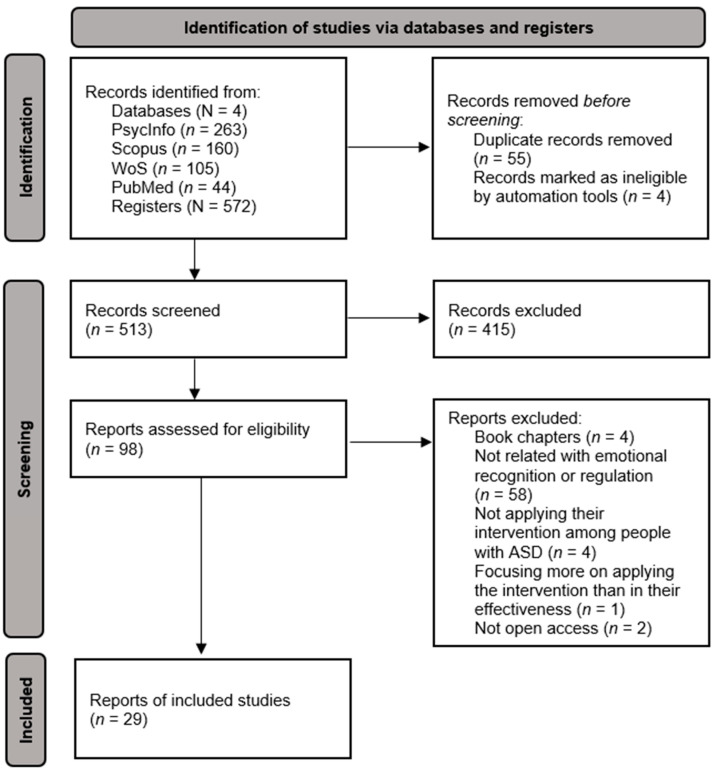
Flow diagram following PRISMA guidelines.

As a result, 263 articles were identified in Psycinfo, 105 in WoS, 160 in Scopus, and 44 in PubMed, with 572 articles in total. This number decreased to 513 after removing the articles that were duplicated. After the initial screening, during which the abstracts of all articles were reviewed, only 98 articles were selected. Of them, 69 were excluded after reading the body of their texts entirely, due to the following reasons: the intervention was not related to ER or recognition (*n* = 58), the intervention was applied to parents and caregivers rather than individuals with ASD (*n* = 4), the document was a book chapter rather than a scientific study (*n* = 4), the article focused more on the development of the intervention rather than its effectiveness and outcomes (*n* = 1), and obtaining access to the articles was not possible (*n* = 2). A total of 29 articles met the criteria to be included in the review (see Figure 1).

Table 1 presents the characteristics of the final studies included in the review, as well as a synthesis of their results, organized chronologically by the year of publication.

### 3.2. Synthesis of Results

#### 3.2.1. Demographic Characteristics

The analyzed studies were mostly conducted in the United States (*n* = 8) and Asia (four in China, two in Japan, two in Israel, one in Taiwan, and one in South Korea). In total, they covered 62.07% of the articles included in the review. On the other hand, in Europe, studies have been carried out in different countries such as Italy (*n* = 2), Macedonia (*n* = 1), Spain (*n* = 1), Sweden (*n* = 1), and the United Kingdom (*n* = 1), representing 20.69% of the total. Additionally, one study was conducted in Morocco and six were conducted in Australia (20.69%).

The sample population is composed of 1,061 children, adolescents, and young adults. Participants between 4 and 13 years of age were considered children, participants between 14 and 18 were considered adolescents, and participants over 18 years of age were considered adults or young adults. The studies focused mainly on children (twenty-one articles), while only three articles focused on adults. On the other hand, five articles focused on populations that include children, adolescents, and adults. Therefore, there were 688 participants aged between 4 and 13 years old, representing 64.36% of the sample population; there were 50 participants aged between 14 and 18 years old, representing 4.68%; and 35.73% (382 participants) were aged 18 years old or older.

Most studies used male populations. The total sample was composed of 706 men participants, compared to 99 women. Still, it is noteworthy that several studies do not provide information on the gender of the participants. It should also be highlighted that the only study where the proportion of women exceeded that of men was the one from Lee et al. (2018), which focused on emotional skills.

Finally, according to the possible comorbidities associated with the diagnosis of ASD, 15 articles did not report this in the body of their text. Other studies (*n* = 8) specifically reported excluding people from their samples when they presented with other disorders in addition to ASD (Chen et al., 2014; Frolli et al., 2022; Kandalaft et al., 2013; Kuroda et al., 2022; Lecciso et al., 2021; Lee et al., 2018; Petrovska & Trajkovski, 2019; Ramirez-Melendez et al., 2022). Some of the studies included samples in which participants presented with other disorders. For instance, Shaffer et al. (2022) and Beaumont et al. (2015) indicated the specific diagnoses that were contemplated. In these cases, there were participants with ADHD, anxiety and depressive disorders, oppositional defiant disorders, obsessive compulsive disorders, etc. Other articles stated that their sample included other conditions, but did not explicitly label them. For example, Elhaddadi et al. (2021) included people that could not talk or read, Tse (2020) included people with emotional and behavioral problems, and Matsuda and Yamamoto (2014) said that their sample was composed of people with a lower mental age than their current age. Also, Kandalaft et al. (2013) pointed out that the presentation of depression, which was being managed, was not an exclusion criterium for them, even when they also indicated that presenting with other disorders was not allowed.

#### 3.2.2. Results Regarding Type and Duration of Interventions

Regarding the objectives in each study, all of them are linked with, at least, one of the facets of EI. These include recognition, understanding, management, and the use of emotions (J. D. Mayer et al., 2008). As can be seen in Table 1, eighteen studies focused mainly on ERA (62.07%) and seven focused on social skills (24.14%). Table 1 explicitly shows how the objectives are linked to the methodology used to achieve them, and also the results that arise from them. In terms of effectiveness, most have shown significant improvements in both ERA and ER, as well as in social skills. According to Elhaddadi et al. (2021), the most frequently improved emotions were happiness, anger, sadness, and surprise. On the other hand, some interventions, such as the use of the DVD “The Transporters” by Williams et al. (2012), showed limited and unsubstantiated improvements. Even so, most interventions proved effective and demonstrated a potential increase in ERA in individuals with ASD.

According to training programs, seven studies used facial recognition (Gev et al., 2016; Rice et al., 2015; Russo-Ponsaran et al., 2015; Lacava et al., 2010; Williams et al., 2012; Yan et al., 2018; Young & Posselt, 2012). Of these seven, four articles worked with the same program, which used the DVD “The Transporters”. The results were highly varied, which may be due to the different countries where it was applied. In Australia, the effectiveness of this program was limited, as it increased ERA skills but did not sustain them over time (Williams et al., 2012; Young & Posselt, 2012). On the other hand, in Israel, the results showed an increase in recognition skills, which was sustained for three months, although no differences were observed in emotional vocabulary (Gev et al., 2016). Finally, in China, the program was effective for both recognition skills and emotional vocabulary (Yan et al., 2018). The other three remaining articles demonstrated positive results, which generalized to other contexts. All three articles highlighted an increase in ERA skills, particularly for primary emotions such as sadness, happiness, anger, disgust, and fear. Indeed, the article by Russo-Ponsaran et al. (2015) also highlighted improvements in emotional expression, particularly anger, disgust, and surprise.

Other articles (*n* = 3) used virtual reality (VR) or augmented reality to intervene with ASD subjects (Chen et al., 2014; Frolli et al., 2022; Kandalaft et al., 2013). The results obtained by these interventions were positive. After application, subjects could better recognize emotions, both facially and vocally. Moreover, after the treatment, they expressed their feelings more frequently. Notably, both the control group and the experimental group (Frolli et al., 2022) increased ERA, but the group with the VR intervention was able to recognize emotions much faster.

Some of the studies (*n* = 4) employed different programs related to social skills and ERA (Beaumont & Sofronoff, 2008; Beaumont et al., 2015; Petrovska & Trajkovski, 2019; Shaffer et al., 2022). Their results showed an increase in ER, the development of social skills, and better recognition and emotional understanding. These results were also reported by the parents and teachers of the subjects undergoing the intervention. Also, music therapy was used in two articles to intervene with ASD (Huang, 2023; Ramirez-Melendez et al., 2022). Their results indicated better emotional interpretation through music and a reduction in emotional dysregulation.

Games were used on three occasions (Doernberg et al., 2021; Elhaddadi et al., 2021; Fridenson-Hayo et al., 2017). Their results demonstrated gains in ERA, as well as a greater accuracy and understanding of emotions such as happiness, anger, sadness, and fear. The use of robots was present in two articles. One used robots as facilitators for the intervention and compared them to the use of people (Yun et al., 2017). The results showed no significant differences, with both groups achieving improved ERA. The second article compared the use of robots with computers (Lecciso et al., 2021). The two technologies achieved positive results, increasing both ERA and the expression of basic emotions.

Socio-emotional skills training was employed in three articles. The results of one of them indicate that people with ASD in school settings increased emotional competencies. However, these outcomes were not generalizable to other contexts (Ratcliffe et al., 2014). On the other hand, the results of the others showed an increase in these socio-emotional skills, particularly in recognizing four emotions: surprise, happiness, anger, and sadness (Lee et al., 2018; Matsuda & Yamamoto, 2014).

The remaining articles (17.24%) employed various other interventions. Guastella et al. (2010) used oxytocin, obtaining beneficial results in ERA. Physical exercise was used in Tse (2020) and achieved a significant improvement in emotional expression in ASD subjects. Another intervention used theater (Corbett et al., 2010), where the authors obtained significant results in the facial identification of emotions. Limited benefits on ER were observed when using cognitive-behavioral therapy (CBT) and were not maintained over time (Kuroda et al., 2022). Lastly, Stichter et al. (2010) used social competence, obtaining a clear improvement in ERA.

Regarding the duration of interventions, the average duration was eight and a half weeks. The socio-emotional skills training by Ratcliffe et al. (2014) was the longest, lasting six months. The intervention with matching-to-sample tasks by Matsuda and Yamamoto (2014) was the shortest, lasting approximately one week. However, there were also single-session interventions, such as in the case of Lacava et al. (2010), where the MindReading program was used.

#### 3.2.3. Assessment Tools

The instruments used for the assessment varied depending on what the studies aimed to measure. The most repeated measures were the following. The first was the Autism Diagnostic Observational Schedule ([ADOS-2]; C. Lord et al., 2015), which appears in multiple studies and is the most used tool to assess the primary symptoms for diagnosing ASD. The second was the Autism Diagnostic Interview—Revised ([ADI-R]; C. Lord et al., 1994), which is a clinical interview that helps clarify suspicions of ASD. Also, the Wechsler Intelligence Scale ([WISC]; Wechsler, 2011) was used, which appears repeatedly across different articles in its various versions (WISC-IV or WISC-V) and is used to measure intelligence. Furthermore, other scales were used to assess the variables that are different from ASD and intelligence. For instance, the following scales were used: the Social Communication Questionnaire ([SCQ]; Bölte et al., 2008), which evaluates social communication, the Social Responsiveness Scale ([SRS]; Beule & Karlovsky, 2020), which measures symptom rigidity, the Infant Neuropsychological Battery ([NEPSY-II]; Korkman et al., 2007), which measures neuropsychological functioning, and the Vineland Adaptive Behavior Scales ([VABS]; Sparrow & Cicchetti, 1989), which assess adaptive behavioral skills.

### 3.3. Quality Assessment Results

Considering the quality of the articles included, 24 of the analyzed studies (82.76%) showed consistency in high-quality evidence. Meanwhile, five studies exhibited moderate-quality evidence. However, their recommendation remains strong (see Table 2). Within the context of the GRADE system, this indicates that the benefits of the intervention clearly outweigh its risks and costs, highlighting general applicability, confidence in its implementation, and sufficiently clear results to justify this recommendation. These findings suggest that most individuals within the specific scope of the articles analyzed are likely to achieve similar outcomes.

## 4. Discussion

The RQ in this study was as follows: How effective are the interventions designed to enhance EI in people with ASD? Therefore, the primary purpose of this study is to contribute to the field of research by providing information about the most effective interventions to enhance the emotional competencies of individuals with ASD. To achieve this, an analysis of the existing scientific literature on EI and ASD was conducted through a search of all articles published between 1980 and 2024. The review of the data analyzed provides a detailed view of the effectiveness of these interventions, highlighting different outcomes.

One of the most positive aspects of the results is the cultural diversity presented in the analyzed studies. This wide representation of countries and cultures contributes to the diversity of the sample. The research spans different countries across four continents: Europe, America, Africa, and Oceania. This wide geographic representation allows for a more global understanding of the interventions and suggests that ASD is not an issue confined to certain countries or cultures but is a disorder with worldwide prevalence (Málaga et al., 2019; WHO, 2023). This finding aligns with previous studies that have also identified ASD as a global phenomenon with relatively similar prevalence across various cultures, reinforcing the idea that the disorder is determined by neurobiological factors rather than exclusively by cultural and socioeconomic conditions (Elsabbagh et al., 2012). Nevertheless, it is worth considering whether these interventions that have proven effective in other countries might be culturally adapted. The literature suggests that culture influences social interactions (Davenport et al., 2018; Golson et al., 2022), which could imply that interventions effective in one context may not be equally effective in another. For instance, in the case of the program “The Transporters”, which has been used in countries like Israel, Australia, and China, the results vary significantly among them. This fact suggests that the way in which certain executive functions, such as ERA, are taught and internalized may be influenced by cultural factors. Therefore, to maximize their effectiveness, it may be necessary to adapt interventions to different cultural contexts.

Another noteworthy aspect is the predominance of children in the sample, as 72.41% of the studies focus on this population. This is understandable, since the diagnosis of ASD is usually made at early ages (Loubersac et al., 2023; Rojas et al., 2019) and early interventions have been shown to be more effective in improving the development of social and emotional skills (Wergeland et al., 2022). Moreover, this is very important as it enables children with ASD to reach their maximum potential and improve their quality of life (Rojas et al., 2019). However, this focus on childhood may limit the application of the results to other life stages, such as adolescence or adulthood, since only 10.43% of the studies include this sample.

A critical aspect is the gender disparity in the samples, as the majority of participants are male (66.54% compared to 9.33%). This shows that the sample is quite unbalanced in terms of gender. This bias may be due to the prevalence of ASD, which is four times higher in males than in females (Montagut et al., 2018). However, recent studies suggest that ASD in females may be underdiagnosed due to differences in symptom presentation, particularly in the social domain, as females tend to mask symptoms to fit in socially (Ferri et al., 2018). This highlights the importance of including more females in future research, as this imbalance in the sample limits the ability to generalize the findings to females with ASD.

Additionally, according to ASD’s comorbidities, only two out of the twenty-nine studies selected explicitly reported them (Beaumont et al., 2015; Shaffer et al., 2022). Recent systematic reviews and meta-analyses have shown that ASD usually presents with comorbid disorders, with ADHD, anxiety, and depression disorders being the most repeated (Bougeard et al., 2024; Micai et al., 2023). Functional neurological disorders have also been considered to be related to ASD lately (Vickers et al., 2024). The lack of the evaluation of samples regarding these disorders is clearly a gap in their studies, which might be addressed in future research.

As previously mentioned, ERA and social skills are the areas most addressed in the analyzed studies. This aligns with the original facets of the definition of EI (J. D. Mayer et al., 2008). Most interventions have proven effective in improving these skills, which is consistent with previous studies indicating that deficits in ERA are a core feature of ASD (Dollion et al., 2022; Harms et al., 2010). Nevertheless, some interventions have not been effective, such as CBT, used in the study by Kuroda et al. (2022), and the “The Transporters” DVD, when used in the study of Williams et al. (2012). The emotions most frequently improved by the interventions include happiness, anger, sadness, and fear, which align with the so-called primary or universal emotions (Cowen et al., 2019). This trend is consistent with previous studies suggesting that basic emotions are more easily recognizable than more complex emotions, such as shame, as they involve a greater understanding of social norms (Ekman, 1999).

One of the most interesting conclusions is that interventions using new technologies such as VR, robots, and computer programs tend to be more effective, achieving faster ERA compared to more traditional interventions such as theater or music therapy. This may be because new technologies allow for greater adaptability and personalization of interventions, offering the possibility of creating controlled environments where subjects can repeatedly practice without the pressure of real social interaction (Lahiri et al., 2014). This aligns with the results of a recent systematic review that addresses the use of technology-based interventions in people with ASD, which, among other things, states that they can be useful for increasing their ER (Sumra & Sumra, 2024). However, although these interventions show promising results, traditional interventions should not be underestimated. Theater, for example, has been proven to be effective in improving emotional identification (Corbett et al., 2010). Also, music therapy has shown improvements in emotional interpretation as well as reductions in dysregulation, highlighting the importance of employing multisensory approaches in interventions (Ramirez-Melendez et al., 2022). Moreover, management interventions such as cognitive-behavioral therapies, physical activity, and stress and anger management programs have been indicated for enhancing ER (Sari et al., 2024). Therefore, while new technologies are a valuable tool, they should not entirely replace traditional approaches, but rather complement them to create a more holistic and effective approach (Yun et al., 2017).

Despite the positive results of most interventions, some have not been completely effective or have failed to maintain the achieved results over time. An example is the case of using the program “The Transporters” in Australia, which, although initially improving ERA, does not sustain these improvements over time (Williams et al., 2012). This underscores the importance of designing interventions that not only improve skills in the moment but also promote the consolidation of these improvements and the generalization of these skills to other contexts. Another study that failed to achieve fully effective results is the use of CBT by Kuroda et al. (2022), which, while improving ER in the short term, does not maintain these improvements after the intervention ends.

### 4.1. Limitations and Future Research

It is important to consider the limitations of the present study. First, there is a clear gender disparity in the samples, limiting the generalization of these results to females and girls with ASD, further reinforcing the idea that such disorders predominantly affect males (Calderoni, 2023). Second, some interventions have shown effectiveness only in the short term (e.g., the study by Kuroda et al., 2022), meaning the results of these interventions are not sustained after a period of application, suggesting that more long-term research is needed to assess the durability of effects. Also, regarding the duration of the interventions, differences between studies can be one of the determining factors in the effectiveness of interventions. Although this does not seem to be the only factor, prolonged treatment may offer more time for practicing and consolidating emotional skills.

Another critical aspect is the sample size, as it was quite limited in some studies, being shorter than five participants in a few cases (Chen et al., 2014; Huang, 2023; Lacava et al., 2010; Matsuda & Yamamoto, 2014). This may reduce the ability to generalize findings to the broader ASD population, especially given the wide range of symptomatology and manifestations within the spectrum (S. S. Kuo et al., 2022). Additionally, including studies that explicitly report comorbid disorders among their sample is fundamental, as they are not only common, but could also be sensitive to gender and age (Bougeard et al., 2024). Additionally, although some of the instruments used in the studies are widely known, such as the WISC (Wechsler, 2011), 62.10% of the analyzed studies, or a total of 18 articles, do not report reliability and validity information for their instruments. This lack of information can compromise the robustness of the results obtained. This is particularly relevant for studies using less common measurement tools or those not validated in different populations, as it limits their replicability. Using PRISMA-COSMIN guidelines would be considered interesting for the future if researchers are willing to specifically analyze the psychometric proprieties of the measurement instruments that have been used for this topic (Elsman et al., 2024). However, this is not the case of the present study. The objective of this study fits the definition of PRISMA guidelines better, which are recommended to be used when analyzing the effects of health interventions (Page et al., 2021). Finally, as it happens in systematic reviews and meta-analyses, publication biases must be considered (Nair, 2019). This means it is probable that only the statistically significant results were published, and consequently, taken into account in this review.

### 4.2. Practical Implications

Regarding the practical applications derived from this study, the results suggest that programs based on ERA, VR, or music therapy can be implemented in educational and therapeutic settings to improve the socio-emotional skills of individuals with ASD. An example of this is the case of socio-emotional skills training by Ratcliffe et al. (2014). Although the results do not generalize to other contexts in addition to the classroom, the results showed an improvement in the skills of individuals with ASD. These interventions offer effective tools to promote the social well-being of this population, enabling mental health and education professionals to adapt these strategies to the specific needs of individuals, thereby personalizing approaches and enhancing their effectiveness. In general terms, the implementation of these interventions is beneficial both educationally and socially, as by working on EI, these interventions can promote social inclusion and facilitate the participation of individuals with ASD in everyday settings, thus contributing to their integration and more accessible development.

## 5. Conclusions

Based on the results, and answering the RQ, it can be stated that interventions conducted on individuals with ASD to address EI and achieve improvements in this area can be effective. The reviewed studies suggest that these interventions are particularly effective in the field of ERA. The use of new technologies such as VR or electronic devices such as robots or computers offers promising results, achieving improvements in various facets of EI in a shorter period. Traditional interventions have also proven effective and should not be dismissed.

Finally, it would be advisable to continue exploring new instruments and approaches to more precisely measure EI in this population to further enhance the effectiveness of interventions. Furthermore, future studies should address the generalization of long-term effects, include more women in samples, and explore other areas of EI, such as ER or the use of emotions to evoke thoughts more simply.

## Figures and Tables

**Table 1:** Information from the selected articles.

Authorship, (Publication Year) Country	Objective	Sample N of People with ASD (Female, Male) Age = Min.–Max. Control Group	ASD Comorbidities	Type of Intervention Duration	Assessment Tools	Results
Beaumont and Sofronoff (2008) Australia	To increase social competence, facial expression, body gesture recognition, and anxiety and anger management strategies	N = 47 (5, 44) 7–11 Yes. Random assignment to intervention group (*n* = 26) and control group (*n* = 23)	NR	Implementation of “The Junior Detective Training Program” for Social Skills. Seven weeks of intervention, one weekly session lasting two hours	WISC-III (α = 0.9), CAST, SSQ-P, ERSSQ (α = 0.89), APE-FE, and APE-PC	Improvements in social skills compared to the control group, sustained for at least 5 months. No significant differences between intervention and control groups in ERA
Corbett et al. (2010) USA	To improve the memory of faces, expression of emotions, and ToM	N = 8 (1, 7) 6–17 No	NR	“SENSE theatre”. A community-based intervention program using theater as a social activity. Three months, three to four days per week	WISC-III, NEPSY, SCQ, and SRS	Significant differences in facial and expression recognition in children post-intervention. This intervention helps individuals with ASD improve facial identification and ToM skills
Guastella et al. (2010) Australia	To measure the improvement of ERA in people with ASD	N = 16 (0, 16) 12–19 No. Youth group (*n* = 11), adult group (*n* = 5). Both received oxytocin and a placebo spray one week after	NR	Administration of intranasal oxytocin in doses. RMET program session 45 min post-administration, effects assessed one week later	DBC and WISC-III	Oxytocin improved ERA in 60% of participants, enhancing ERA in young people with ASD
Lacava et al. (2010) USA	To increment ERA and social interaction skills	N = 4 (0, 4) 7–11 No	NR	“MindReading” program. One session lasting 45 to 75 min	CAST, MindReading: CAM-C and RME-C	All participants improved ERA post-intervention in both image-based and computer-based tasks
Stichter et al. (2010) USA	To gain abilities in facial expression recognition, ToM, social abilities, and executive functioning	N = 29 (29, 0) 11–14 No	NR	Intervention based on social competence. Two weeks of intervention	SRS, DANVA-2-CF, BRIEF, ToM, RMET, ADOS, and ADI-R	Participants showed increased recognition of emotional states in children from various images, as well as improved ability to recognize emotional/mental states through eye contact
Young and Posselt (2012) Australia	To improve ERA and understanding, as well as other areas of social impairment	N = 25 (NR) 4–8 Yes. Group 1 (*n* = 13) watched “The Transporters.” Group 2 (*n* = 12) watched “Thomas and Friends”	NR	Watching “The Transporters”. Three weeks of intervention	Face Tasks, NEPSY-II, WPPSI-III, SCQ, and WISC-IV	Children who watched “The Transporters” improved their ability to identify facial expressions and social behavior. The latter was also observed in children who watched “Thomas and Friends.” Both groups showed increased social interest among peers
Kandalaft et al. (2013) USA	To explore the increase in social perception, ERA, ToM, and social conversation	N = 8 (2, 6) 18–26 No	No. Except they presented managed depression	Use of VR. Two weekly sessions lasting one hour over two weeks	ADOS, VR-SCT, WASI, ToM, RMET, ACS-SP (α = 0.75), and SSPA (α = 0.65)	Results showed improved ERA over emotions in faces and voices post-intervention
Williams et al. (2012) Australia	To determine the improvement in ERA, ToM, and social skills	N = 55 (7, 48) 4–7 Yes. Group 1 (*n* = 28) watched “The Transporters.” Group 2 (*n* = 27) watched “Thomas and Friends”	NR	Watching “The Transporters”. Three months of intervention	ADOS, VABS, WPPSI-III, NEPSY-II, and emotional recognition and matching tasks	Limited effectiveness: while participants improved their ability to identify anger, this was not sustained over time. ERA improvements did not generalize to other life settings
Chen et al. (2014) Taiwan	To increase the ERA and social skills among people with ASD	N = 3 (0, 3) 10–13 No	No	ARSFM. Seven sessions of approximately one hour each	WISC-IV and ARSFM	The participants increased their social skills and tried more frequently to express their own feelings after the treatment. In addition, they also recognize and respond more appropriately to the emotional expressions they encounter on a daily basis
Matsuda and Yamamoto (2014) Japan	To acquire the recognition of facial expressions based on movies of socio-emotional situations	N = 2 (0, 3) 4–8 No	Verbal and mental ages were lower than their current ages	Training with MTS through the use of films and images of social-emotional situations. One week with daily sessions of 15 to 20 min	CARS, PVT-R, WISC-IV, and the MTS program itself	Both children increased their recognition of four emotions: happiness, surprise, anger, and sadness. Therefore, their percentage of correct answers increased, and in addition, these ERA results generalized to other areas
Ratcliffe et al. (2014) Australia	To increase emotional competency, social skills, and mental health	N = 217 (22, 195) 8–14 Yes. 106 students were in the intervention group, along with their parents and teachers, and 111 students were in the control group	NR	EBSST. 16 sessions of 90 min each divided into three modules over 6 months	EBSST (α = NR), EDQ (α = 0.92), SRS (α = 0.97), SSIS-RS (α = 0.70), and SDQ (α = 0.73)	Teachers reported that there was an increase in emotional competencies in the group that received the treatment, while parents did not report the same increases at home, suggesting that the results do not generalize
Beaumont et al. (2015) Australia	To improve social skills, ER, and behavior in school and home	N = 69 (5, 64) 7–12 Yes. Group 1, structured intervention (*n* = 35) and group 2, unstructured intervention (*n* = 34)	Yes (*n* = 16) ADHD, anxiety, depression, oppositional defiant disorder, sensory processing disorder, and speech and language impairments	SAS program. 10 weeks of intervention with one 90 min session per week (or two 45 min sessions per week)	WASI, SSQ-P-T (α = 0.92), ERSSQ-P-T (α = 0.90), SCAS-P (α = 0.95), and CAPES-DD (α = 0.87)	According to teacher reports, both groups improved significantly in ER and social skills, with greater improvements in the structured intervention group
Rice et al. (2015) USA	To examine the increase in affect and facial recognition, mentalizing and positive interactions with peers, as well as the decrease in social impairment and negative interactions with peers	N = 31 (3, 28) 5–11 Yes. Control group (*n* = 15) and experimental group (*n* = 16)	NR	FaceSay^TM^ program. 25 min of intervention per week for 10 weeks	NEPSY.II (α = 0.69), FaceSay^TM^, SucessMaker, SRS-2, and WISC-IV	Participants increased their ability to recognize basic emotions such as happiness, sadness, anger, disgust, and fear
Russo-Ponsaran et al. (2015) USA	To improve facial ERA and expression	N = 25 (3, 22) 8–15 Yes. Control group (*n* = 13) and experimental group (*n* = 12)	NR	MiX^TM^ emotional recognition training Two one-hour sessions per week for 8 weeks	WASI-2, ADI-R, WISC-IV, VABS, DSAS, SCQ, ADOS, MiX^TM^, CATS, NEPSY-II, CASP, DANVA, and CELF-4	Significant improvements in facial emotion recognition, improvements in participants’ ability to express facial emotions, especially anger, disgust, and surprise, and generalization of these skills to broader contexts
Gev et al. (2016) Israel	To examine the improvement and maintenance of ERA and emotional vocabulary	N = 59 (50, 9) 4–7 Group 1. Transporters intervention with parental support (*n* = 15). Group 2. Transporters intervention without parental support (*n* = 14). Group 3. Control group with parental support (*n* = 16). Group 4. Control group without parental support (*n* = 14)	NR	Watching “The Transporters”. Eight weeks of intervention	ADOS-2, WPPSI, VABS, CAST y ER tasks, and emotion vocabulary task	The results showed an increase in ERA skills in preschoolers with high-functioning ASD, and these are maintained after three months. On the other hand, there are no differences in emotional vocabulary between those who watched The Transporters DVD and those who did not, and having or not having parental support did not affect ERA either
Fridenson-Hayo et al. (2017) Israel, UK, and Sweden	To improve ERA	Israel: N = 43 (NR) the UK: N = 15 (NR) Sweden: N = 40 (NR) NR No	NR	EmotiPlay program. Eight weeks of intervention	Recognition tasks, WISC-IV, SRS-2, ADOS-2, and VABS-2	Results showed significant gains in tasks related to ERA, influencing three different cultures
Yun et al. (2017) South Korea	To evaluate the effects of the intervention among eye contact and facial ERA	N = 15 (NR) 4–7 Yes. Experimental group, with robot (*n* = 8), and control group, without robot (*n* = 7)	NR	Use of a robot as a facilitator. Eight sessions of about 30–40 min	ADOS, ADI-R, VABS, SCQ, SRS, CBCL, and eye contact frequency through recordings	In both groups, correct responses to recognize emotions in faces increased after the intervention, as well as increased eye contact. The differences were not significant
Lee et al. (2018) China	To intervene in increasing ERA within and without context, emotion expression, and seeking help and techniques for ER	N = 8 (6, 2) 6–9 No	No	Intervention based on emotional skills. One session per week for 10 weeks	CARS (α = 0.74), WISC-IV (α = 0.67), BERS (α = 0.91), and VABS (α = 0.94)	Following the intervention, the children’s emotional and behavioral skills as well as their adaptive communication increased significantly. The largest significant increases were seen in the areas of school and intrapersonal functioning
Yan et al. (2018) China	To demonstrate improvements in ERA	N = 21 (3, 18) 4–6 Yes. Experimental group with ASD (*n* = 7), control group with ASD (*n* = 7), and control group without ASD (*n* = 7)	NR	Watching “The Transporters”. Six weeks. Sessions of 40 min, five days per week	WISC-IV and emotional vocabulary and facial expressions and emotional situation tasks	The use of the DVD “The Transporters” is effective in the intervention, increasing their skills and emotional vocabulary
Petrovska and Trajkovski (2019) Macedonia	To enhance emotional understanding	N = 32 (10, 22) 7–15 Yes. Experimental group (*n* = 16) and control group (*n* = 16)	No	Ucime Emocii program with professional supervision. Eight weeks of intervention	CARS (α = 0.89), ECT, and face and situation task	Positive and significant effects were observed in ERA and understanding, with the experimental group showing a significant improvement
Doernberg et al. (2021) USA	To improve emotional understanding abilities and cognitive and affective play skills	N = 25 (3, 22) 6–9 Yes. Another sample that did not play these games was used as a control group	NR	Use of simulation games. Five sessions of 20 min per week	APS (α = 0.80), KAI-R (α = 0.80), ADOS, and ADI-R	Children in the experimental group (*n* = 25, those who played the simulation games) increased their imagination score, which is directly related to their empathic skills, a crucial factor in emotional understanding. They also significantly improved their emotional understanding, especially in describing their own experiences when they felt happy, sad, angry, scared, afraid, in love, proud, guilty, jealous, anxious, or lonely
Tse (2020) China	To examine the impact on ER and behavioral functioning	N = 27 (12, 13) 8–12 Yes. Experimental group (*n* = 15) and control group (*n* = 12)	Presence of severe emotional or behavioral problems indicated by CBCL	Use of physical exercise. 48 sessions, four sessions per week of 30 min each	SRS-2, WISC-IV, ERC (α = 0.70), and CBCL (α = 0.76)	The results showed a significant improvement in emotional expression after physical exercise. Exercise increases general mood as well as self-awareness
Elhaddadi et al. (2021) Morocco	To improve social skills and emotional social interactions	N = 32 (8, 24) 6–14 No	Some of them did not have the ability to read (*n* = 14) or the ability to speak (*n* = 10)	JeStiMulE game. Four weeks with two one-hour sessions per week	Raven’s progressive matrices, the Rimland E2 questionnaire, a face test, and JeStiMulE itself	After the intervention, children were more accurate in recognizing emotions. In fact, they were much better at recognizing facial expressions of happiness, anger, sadness, and fear, compared to other emotions such as pain and surprise
Lecciso et al. (2021) Italy	To explore the increment in ERA and expression abilities	N = 12 (0, 12) 6–13 No. Robot intervention (*n* = 6) and computer intervention (*n* = 6)	No	Hybrid training based on the comparison between intervention with robots and intervention with computers. Four days of intervention plus a pre- and post-intervention period to analyze the results	Raven’s colored progressive matrices, ADOS, ADI-R, FERT, and BEPT	There were no significant differences between the human robot intervention and the computer intervention in recognizing and expressing emotions. All children increased their ability to recognize basic emotions when trained by electronic devices and also expressed their basic emotions better
Frolli et al. (2022) Italy	To improve the ability to recognize and label emotions, as well as linking them to situations	N = 60 (10, 50) 9–10 Yes. Control group, traditional intervention with therapist (*n* = 30), and experimental group, intervention using VR (*n* = 30)	No	Use of VR. Three months, three times per week on different days	ADOS, WISC-IV, SES, and K-SADS-PL	Both groups acquired primary emotion recognition skills in a similar way, but the group receiving VR intervention acquired secondary skill recognition faster
Kuroda et al. (2022) Japan	To acquire ER strategies, increase emotional awareness (both their own and that of others), and improve their knowledge about ASD	N = 58 (NR) 18–50 Yes. Experimental group (*n* = 29) and control group (*n* = 29)	No	Cognitive-behavioral therapy program. Each weekly session lasted approximately 100 min for eight weeks	ADOS, ADI-R, WAIS-III, AQ, SRS-A, SCQ, CISS, TAS20, and ESQ	The results showed how the experimental group increased their ability to describe feelings and their emotional regulation strategies. Despite these findings, 12 weeks after the intervention, they were not maintained, so their clinical usefulness is limited
Ramirez-Melendez et al. (2022) Spain	To improve ERA and facial expression	N = 25 (0, 25) 6–11 Yes. Experimental group (*n* = 14) and control group (*n* = 11)	No	Use of music as a tool for intervention. Four weeks.	ADOS, Emotiv EPOC© EEG system, and WISC-IV	The experimental group showed a significant effect on emotional interpretation at the end of the study. There are no significant results in the conditions in which verbal responses are given without music
Shaffer et al. (2022) USA	To help adjust emotional experience, expression, and intensity to the context	N = 44 (NR) 8–18 No	Yes. ADHD (*n* = 27), anxiety disorders (*n* = 26), obsessive compulsive disorder (*n* = 11), depressive disorder (*n* = 10), oppositional defiant disorder (*n* = 9), intermittent explosive disorder (*n* = 8), insomnia (*n* = 7), and post-traumatic stress disorder (*n* = 3)	“Regulating Together” program. Two weekly sessions of 90 min each for five weeks	VABS, ADOS-2, CGI-S, and WASI-II	The use of this program increased emotional regulation. Not only do subjects with ASD themselves show regulation skills, but caregivers themselves report less dysregulation
Huang (2023) China	To help express emotions, needs, communicate with language, and control the emotional behavior	N = 4 (NR) 4–5 No. Two children were part of the Orff music group, and two others were part of the visual music group	NR	Music therapy. Four months of intervention, one class in the morning and one class in the afternoon, both 45 min long, each day	Observation and Evaluation Form of Autism Children’s Emotional Disorder	Different types of music therapy have different effects on emotional disorders. Orff music is a combination of voices, dances, languages, and rhythms. Visual music is a way of combining sound with an image in rhythm. Both styles reduce the occurrence of emotional dysregulation. On the other hand, visual music is better for those who present less emotional disorders

Note. ASD: Autism Spectrum Disorder; NR: Not reported; WISC: Wechsler Intelligence Scale; CAST: Childhood Asperger Syndrome Test; ERSSQ: Emotion Regulation and Social Skills Questionnaire; APE-FE: Assessment of Perception of Emotion from Facial Expression; APE-PC: Assessment of Perception of Emotion from Posture Cues; ERA: Emotional Recognition Ability; ToM: Theory of Mind; NEPSY: Infant neuropsychological battery; SCQ: Social Communication Questionnaire; SRS: The Social Responsiveness Scale; DBC: Developmental Behavior Checklist; ASD: Autism Spectrum Disorder; CAM-C: Cambridge Mindreading Face-Voice for Children; RME-C: Reading The Mind in the Eyes, Child version task; DANVA-2-CF: The Diagnostic Analysis of Non-Verbal Accuracy-2, Child Facial Expression; BRIEF: The Behaviour Rating Inventory of Executive Function; ADOS: Autism Diagnostic Observational Schedule; ADI-R: Autism Diagnostic Interview—Revised; WPPSI: Wechsler Preschool and Primary Scale of Intelligence; VR: Virtual reality; VR-SCT: Virtual Reality Social Cognition Training; WASI: Wechsler Abbreviated Scale of Intelligence; ACS-SP: Advanced Clinical Solutions for WAIS-IV Social Perception Subtest; SSPA: Social Skills Performance Assessment; VABS: Vineland Adaptative Behaviour Scale; ARSFM: Augmented Reality-Based Self-Facial Modeling; MTS: Matching-to-sample; CARS: Childhood Autism Rating Scale; PVT: Psychomotor Vigilance Task; EBSST: Emotion-Based Social Skills Training; EDQ: Emotions Development Questionnaire; SSIS-RS: Social Skills Improvement System—Rating Scales; SDQ: Strengths and Difficulties Questionnaire; ER: Emotional Regulation; ADHD: Attention-Deficit/Hyperactivity Disorder; SAS: Secret Agent Society; SSQ-P: Social Skills Questionnaire; SCAS-P: The Spence Children’s Anxiety Scale-Parents Version; CAPES-DD: Child Adjustment and Parent Efficacy Scale-Developmental Disability-Parent; DSAS: Dishion Social Acceptance Scale; CATS: Child and Adolescent Trauma Screen; CASP: The Child and Adolescent Scale of Participation; CELF-4: Clinical Evaluation of Language Fundamentals; CBCL: Child Behaviour Checklist; BERS: Behavioural and Emotional Rating Scale; ECT: Emotion Comprehension Test; APS: The Affect in Play Scale; KAI-R: The Kusche Affective Inventory Revised; ERC: Emotion Regulation Checklist; FERT: Facial Expression Recognition Test; BEPT: The Basic Emotion Production Task; SES: Effects of Socioeconomic Status; K-SADS-PL: Schedule for Affective Disorders and Schizophrenia; AQ: Autism Spectrum Quotient; CISS: Coping Inventory for Stressful Situations; TAS20: Toronto Alexithymia Scale; ESQ: Executive Skills Questionnaire; EEG: Electroencephalography; CGI-S: Clinical Global Impressions Scale.

**Table 2:** GRADE Scale results.

Study	Recommendation Grade	Evidence Level
Beaumont and Sofronoff (2008)	1A	A
Corbett et al. (2010)	1B	B
Guastella et al. (2010)	1A	A
Lacava et al. (2010)	1B	B
Stichter et al. (2010)	1A	A
Young and Posselt (2012)	1A	A
Kandalaft et al. (2013)	1A	A
Williams et al. (2012)	1A	A
Chen et al. (2014)	1B	B
Matsuda and Yamamoto (2014)	1B	B
Ratcliffe et al. (2014)	1A	A
Beaumont et al. (2015)	1A	A
Rice et al. (2015)	1A	A
Russo-Ponsaran et al. (2015)	1A	A
Gev et al. (2016)	1A	A
Fridenson-Hayo et al. (2017)	1A	A
Yun et al. (2017)	1A	A
Lee et al. (2018)	1A	A
Yan et al. (2018)	1A	A
Petrovska and Trajkovski (2019)	1A	A
Doernberg et al. (2021)	1A	A
Tse (2020)	1A	A
Elhaddadi et al. (2021)	1A	A
Lecciso et al. (2021)	1A	A
Frolli et al. (2022)	1A	A
Kuroda et al. (2022)	1A	A
Ramirez-Melendez et al. (2022)	1A	A
Shaffer et al. (2022)	1A	A
Huang (2023)	1B	B

Note. A: High-quality evidence, B: moderate-quality evidence, 1: strong recommendation.

## Data Availability

No new data were created or analyzed in this study. Data sharing is not applicable to this article.

## Abbreviations

The following abbreviations are used in this manuscript:

WHO	World Health Organization
ASD	Autism Spectrum Disorder
DSM	Diagnostic and Statistical Manual of Mental Disorders
APA	American Psychological Association
ToM	Theory of Mind
ID	Intellectual Disability
IQ	Intelligence Quotient
EI	Emotional Intelligence
ER	Emotional Regulation
ERA	Emotional Recognition Ability
PICO	Patients/Problem, Intervention, Comparison, Outcomes
RQ	Research Question
PRISMA	Preferred Reporting Items for Systematic Reviews and Meta-Analyses
PROSPERO	International Prospective Register of Systematic Reviews
WoS	Web of Science
GRADE	Grading of Recommendations Assessment, Development, and Evaluation
ADHD	Attention-Deficit/Hyperactivity Disorder
VR	Virtual Reality

## Appendix A

**Table A1:** PRISMA checklist indicating where to find each item.

Item	Yes	No	Page	NA
TITLE				
1. Title	X		1	
ABSTRACT				
2. Abstract	X		1	
INTRODUCTION				
3. Rationale	X		1	
4. Objectives	X		2	
METHODS				
5. Eligibility criteria	X		3	
6. Information sources	X		2	
7. Search strategy	X		2	
8. Selection process	X		3	
9. Data collection process	X		3	
10. Data items	X		3	
11. Bias assessment	X		3	
12. Effect measures				X
13. Synthesis methods				X
14. Reporting bias assessment				X
15. Certainty assessment				X
RESULTS				
16. Study selection	X		4	
17. Study characteristics	X		4	
18. Risk of bias	X		19	
19. Individual studies	X		4	
20. Synthesis	X		17	
21. Reporting biases				X
22. Certainty of evidence				X
DISCUSSION				
23. Discussion	X		19	
OTHER				
24. Registration	X		2	
25. Support	X		23	
26. Competing interests	X		23	
27. Available data	X		23	

Note. NA: Not applicable.
